# Separate hydrolysis and fermentation of softwood bark pretreated with 2-naphthol by steam explosion

**DOI:** 10.1186/s13068-024-02552-y

**Published:** 2024-07-17

**Authors:** Andreas Averheim, Stefan Stagge, Leif J. Jönsson, Sylvia H. Larsson, Mikael Thyrel

**Affiliations:** 1 Fiber Technology Center, Valmet AB, 851 94 Sundsvall, Sweden; 2 Department of Chemistry, Umeå University, 901 87 Umeå, Sweden; 3 Department of Forest Biomaterials and Technology, Swedish University of Agricultural Sciences, 901 83 Umeå, Sweden

**Keywords:** Steam explosion, Softwood bark, 2-Naphthol, Enzymatic hydrolysis, Fermentation, Inhibition

## Abstract

**Background:**

2-Naphthol, a carbocation scavenger, is known to mitigate lignin condensation during the acidic processing of lignocellulosic biomass, which may benefit downstream processing of the resulting materials. Consequently, various raw materials have demonstrated improved enzymatic saccharification yields for substrates pretreated through autohydrolysis and dilute acid hydrolysis in the presence of 2-naphthol. However, 2-naphthol is toxic to ethanol-producing organisms, which may hinder its potential application. Little is known about the implications of 2-naphthol in combination with the pretreatment of softwood bark during continuous steam explosion in an industrially scalable system.

**Results:**

The 2-naphthol-pretreated softwood bark was examined through spectroscopic techniques and subjected to separate hydrolysis and fermentation along with a reference excluding the scavenger and a detoxified sample washed with ethanol. The extractions of the pretreated materials with water resulted in a lower aromatic content in the extracts and stronger FTIR signals, possibly related to guaiacyl lignin, in the nonextractable residue when 2-naphthol was used during pretreatment. In addition, cyclohexane/acetone (9:1) extraction revealed the presence of pristine 2-naphthol in the extracts and increased aromatic content of the nonextractable residue detectable by NMR for the scavenger-pretreated materials. Whole-slurry enzymatic saccharification at 12% solids loading revealed that elevated saccharification recoveries after 48 h could not be achieved with the help of the scavenger. Glucose concentrations of 16.9 (reference) and 15.8 g/l (2-naphthol) could be obtained after 48 h of hydrolysis. However, increased inhibition during fermentation of the scavenger-pretreated hydrolysate, indicated by yeast cell growth, was slight and could be entirely overcome by the detoxification stage. The ethanol yields from fermentable sugars after 24 h were 0.45 (reference), 0.45 (2-naphthol), and 0.49 g/g (2-naphthol, detoxified).

**Conclusion:**

The carbocation scavenger 2-naphthol did not increase the saccharification yield of softwood bark pretreated in an industrially scalable system for continuous steam explosion. On the other hand, it was shown that the scavenger's inhibitory effects on fermenting microorganisms can be overcome by controlling the pretreatment conditions to avoid cross-inhibition or detoxifying the substrates through ethanol washing. This study underlines the need to jointly optimize all the main processing steps.

## Background

Since the introduction of the carbocation scavenger 2-naphthol in a pretreatment process to enhance subsequent enzymatic hydrolysis in 2015 [1], several authors have investigated scavenger pretreatments. Feedstocks such as poplar [1–4], spruce [1, 4–6], pine [4, 7, 8], larch [9], bamboo [10], birch [7], and beech [4] have been studied, confirming the enhancing potential of adding 2-naphthol to the pretreatment. On the other hand, the toxicity of 2-naphthol to fermentation organisms [6] must be adequately investigated and addressed if feasible process concepts are to be presented.

The inclusion of 2-naphthol presumably counteracts the condensation of lignin during the acidic processing of lignocellulosic biomasses, such as hydrothermal, steam explosion, or dilute acid pretreatments. These pretreatments have been widely researched for biomass conversion into biofuels, e.g., second-generation bioethanol via enzymatic hydrolysis and fermentation, renewable platform chemicals, or biomaterials [11]. Apart from depolymerizing hemicellulose [11], the processing of lignocellulosic materials under these acidic conditions affects acid-labile structures in lignin, especially by the cleavage of β-O-4 linkages followed by instant repolymerization reactions to form condensed C–C structures [12, 13]. The reactions proceed via a carbocation intermediate. Thus, a nucleophilic reagent, such as 2-naphthol, acts as a carbocation scavenger and, in that way, prohibits condensation reactions [1, 12, 14].

The repolymerization of lignin is considered an obstacle to the enzymatic conversion of pretreated biomass and one of the main factors behind softwood recalcitrance [4]. Condensed lignin may act as a physical barrier [2], but more importantly, it promotes nonproductive binding and deactivation of the enzyme [2, 3, 8, 9]. Mitigated lignin condensation by the addition of 2-naphthol during pretreatment can increase biomass porosity and decrease lignin surface coverage, thus diminishing steric hindrance [2]. It has been proposed that the nonproductive binding of enzymes can be minimized due to reduced inhibitory phenolics [3, 8] and decreased lignin surface area [1]. Interestingly, it has also been discovered that pretreatment with lignins derived from 2-naphthol treatments may promote the activity of lytic polysaccharide monooxygenases present in modern enzyme cocktails, consequently promoting the oxidative depolymerization of cellulose [5].

Phenolic compounds may inhibit the fermentation of saccharides into ethanol with baker's yeast *Saccharomyces cerevisiae* [15]. Although pretreatments with carbocation scavengers have been extensively studied in combination with enzymatic treatments, information regarding the fermentability of broths in the presence of the phenolic compound 2-naphthol is scarce. Seidel et al. [6] conducted fermentations using two-stage pretreated softwood as a substrate, including washed and unwashed substrates, at various solid loadings. The study showed that whole-slurry fermentation at 10% solids loading, which could be considered relevant for industrial applications [16, 17], was severely inhibited by the presence of 2-naphthol. The threshold concentration for inhibition was lower than that in more dilute systems, indicating cross-inhibition between 2-naphthol and degradation products from hemicelluloses, such as acetic acid, furfural, and 5-hydroxymethylfurfural (5-HMF) [6].

This investigation aimed to study the effect of 2-naphthol addition on softwood bark pretreated via continuous steam explosion prior to enzymatic saccharification followed by fermentation using *Saccharomyces cerevisiae*. The enzymatic saccharification and fermentation steps were kept apart in a separate hydrolysis and fermentation (SHF) setup, to facilitate stepwise analysis of the 2-naphthol effects. This approach differs from previous studies regarding the substrate, pretreatment approach, and analyses of the effects of the inclusion of 2-naphthol. In addition, a simple detoxification procedure was tested to study whether any potential inhibitory effects could be easily overcome.

## Methods

### Raw material

The raw material was softwood bark collected at a pulp mill along the northern coast of Sweden. The material was downsized by coarsely shredding it over a 30-mm sieve followed by screening through a 14-mm mesh screen. High-density particles such as sand and gravel were removed before the steam explosion pretreatment to ensure stable operation. The bark consisted of a mixture of Norway spruce (*Picea abies* Karst. L.) and Scots pine (*Pinus sylvestris* L.) with a total solids content of 41%. Further details regarding the sampling and preprocessing procedures are reported elsewhere [18].

### Steam explosion pretreatment

**Fig. 1:**
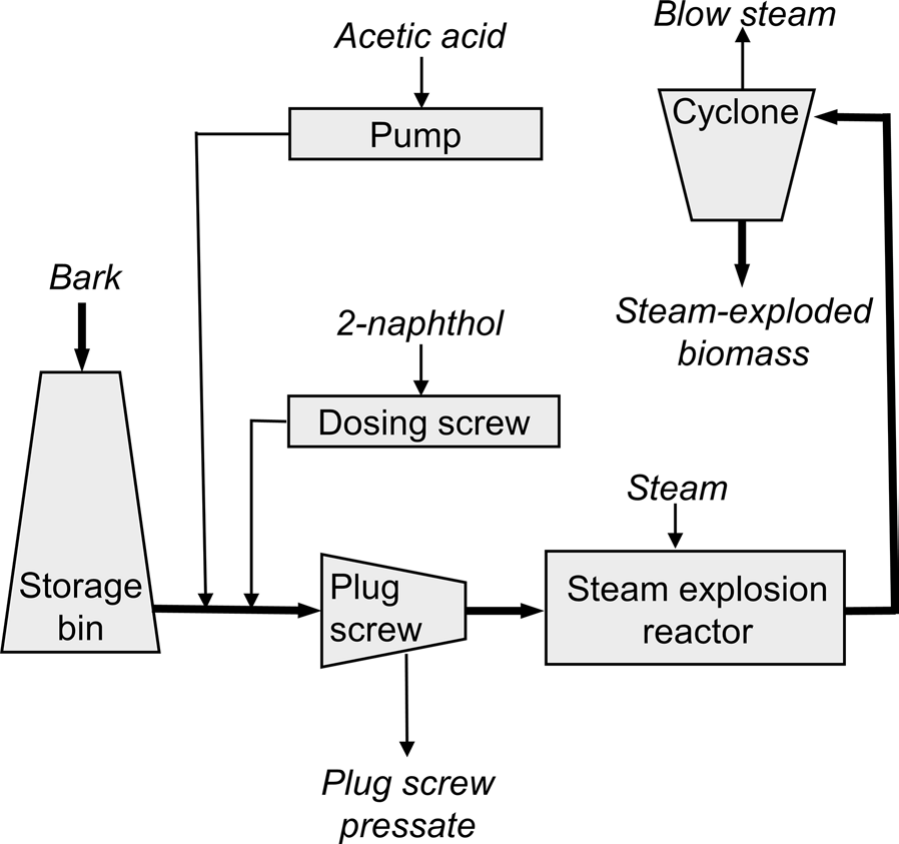
Schematic illustration of the steam explosion system

The bark was pretreated in a pilot system for continuous steam explosion (Valmet BioTrac, Valmet AB, Sundsvall, Sweden), as detailed in the literature [18]. A feed rate of approximately 60 kg h^−1^ on a dry basis, a reactor temperature of 200 °C (corresponding to 14.5 barg steam pressure), and a residence time of 10 min were applied. The chemicals, 98–99% glacial acetic acid (Swed Handling AB, Sweden) diluted to 2.5% (w/v) and 98% 2-naphthol (Thermo Fisher Scientific) milled in a knife-mill (Retsch SM 200) to a particle size < 2 mm were added to the biomass in the atmospheric part of the pretreatment system according to the schematic illustration in Fig. 1. An even and continuous dosage of chemicals and biomass, together with tumbling of the mixture in two conveyor screws before entering the plug screw, ensured the mixing of the chemicals with the biomass. Two steam explosion conditions were applied using 0 and 5% (w/w) 2-naphthol addition, each with 0.5% (w/w) acetic acid dosage based on the total solid content of the biomass feed.

Samples from tests with 2-naphthol (2N) and without (Ref) were taken directly from the steam explosion system outlet and stored frozen before further analysis. In addition, a third sample was produced by detoxifying the sample treated with 2-naphthol (2N EW). The sample was mixed with ethanol at ambient temperature at a ratio of 1:3.2 (w/w) for 15 min, after which the ethanol was decanted. This procedure was repeated three times. The ethanol was then removed through filtering, after which the final undissolved residue was evaporated at ambient temperature under a fume hood.

### Substrate characterization

#### Determination of furans, total aromatics, and total phenolic content in hydrolysates

To obtain the hydrolysates, the substrates were first diluted from ~ 40% to 20% total solids with boiling water. The slurries were kneaded in a kitchen mixer (Bosch MUM8) at the low-speed setting for 15 min, and hydrolysates were then extracted by mechanical compression of the slurries through a wire cloth. The total aromatic content (TAC) of the liquids was measured by UV‒Vis absorption at 280 nm [19] in 96-well microplates using an Epoch™ 2 spectrophotometer and Gen 5™ ver. 1.10 software (BioTek Instruments, Inc., USA, as described in Wang et al. 2018). TAC values will cover heteroaromatics, such as furans, and aromatics, such as phenolic substances. The main furans, furfural, and 5-HMF were analyzed separately using a Dionex UltiMate 3000 HPLC system (Thermo Fisher Scientific, Waltham, MA, USA) as described previously [20, 21]. The total aromatic content, excluding the furans, could then be calculated by accounting for their absorbance at 280 nm by running a blank sample with corresponding 5-HMF and furfural concentrations.

The Folin–Ciocalteu assay was used to determine the total phenolic content [22]. Twenty µL of each sample and a series of vanillin standards at concentrations ranging from 0–300 mg/L were incubated with tenfold dilutions of Folin–Ciocalteu reagent (Merck) for 5 min. Eighty µL of 1 M sodium carbonate solution was added to all the samples. The samples were mixed thoroughly and kept at ambient temperature for 45 min. Afterwards, the UV‒Vis absorption at 760 nm was determined using 96-well microplates, and the total phenolic content was calculated based on vanillin standards.

#### Solvent extraction of pretreated materials

The extracts were removed from the pretreated materials by extraction with cyclohexane/acetone (9:1 v/v) for nonpolar extractives and then with ethanol for polar extractives in a Dionex ASE 350 system (Dionex, Sunnyvale, CA). The nonextractable solids were dried in a fume hood between extractions to allow complete evaporation of the solvents. The extract yield was determined for each extraction by evaporating the solvent and drying at 105 °C overnight.

Cyclohexane/acetone extraction was carried out at 140 °C and 100 bar pressure for four cycles, while the second extraction with ethanol was conducted at 80 °C for eight cycles. The cycle time was eight minutes per cycle, and the rinse volume was 150%. The nonextractable solids were dried in a fume hood and transferred to fresh cells for ethanol extraction. The second extraction was conducted at 80 °C for eight cycles with a residence time of 8 min each and a rinse volume of 150%.

After the two-stage extraction, the nonextractable solids were subjected to acid hydrolysis to determine the acid-insoluble lignin (Klason lignin) content [23].

#### Nuclear magnetic resonance spectroscopy analysis

Samples for ^1^H nuclear magnetic resonance (NMR) spectroscopy were prepared from the dried cyclohexane/acetone extracts by dissolving approximately 20 mg of each sample in 600 μL of acetone-d_6_. The spectra were recorded on a Bruker 600 MHz Avance III HD spectrometer equipped with a BBO cryo-probe. Eight scans were recorded with a relaxation delay of 1 s and a sweep width of 20 ppm.

^13^C cross-polarization magic angle spinning (CP-MAS) NMR spectroscopy was performed on samples from the solid nonextractable fractions after cyclohexane/acetone extraction. A Bruker 500 MHz Avance III spectrometer operating at a ^13^C frequency of 125.75 MHz and equipped with a 4 mm MAS probe was used. Approximately 80 mg of each sample was transferred, as a dry powder, into a 4 mm ZrO_2_ rotor. A 1 ms contact time was used, with a ramped ^1^H pulse amplitude (50–100%). Spinal64 ^1^H decoupling was applied during the acquisition time, and 8192 scans were accumulated for each spectrum at a spin rate of 10 kHz. Adamantane was used as an external chemical shift reference.

CP-MAS spectra were recorded at ambient temperature, and ^1^H spectra were recorded at 298 K. All spectra were processed in Topspin 3.6 (Bruker Biospin, Germany).

#### Fourier transform infrared spectroscopy analysis

Samples for Fourier transform infrared spectroscopy (FTIR) were prepared by washing the steam-exploded materials extensively with 20 °C tap water, drying them without forced convection at 45 °C, and milling them with a knife-mill (IKA A11 basic, IKA-Werke GmbH & Co) to < 0.5 mm. The samples were mixed with IR spectroscopy grade KBr and manually ground in an agate mortar and pestle, and their spectra were measured [24]. Spectra in the range of 4000 to 400 cm^−1^ with a resolution of 4 cm^−1^ were recorded on a Bruker IFS 66v/S spectrometer (Bruker Corporation). 128 scans were coadded for background (pure KBr) and sample and collected in OPUS version 5 software (Bruker Corporation). The data were then processed using MATLAB R2021b (MathWorks) with the open-source graphical user interface available from the Vibrational Spectroscopy Core Facility at Umeå University [25]. The spectral range was cut to the fingerprint region 500–1850 cm^−1^, and baseline correction was performed using asymmetrical least squares [26] with λ = 100 000 and p = 0.001. Finally, total area normalization without curve smoothing was conducted in the cut spectral range.

#### Carbohydrate composition

The carbohydrate compositions of the pretreated materials were measured according to the laboratory analytical procedures available from National Renewable Energy Laboratory. Sugar concentrations were determined with an HPAEC system, Dionex ICS-6000, with CarboPac SA-10 guard and analytical columns. The content of monomeric sugars in the hydrolyzate was subtracted from the total amount of saccharides in the pretreated materials to obtain the total amount of poly- and oligosaccharides.

### Enzymatic saccharification

The steam-exploded materials were subjected to SHF treatments. Enzymatic hydrolysis was performed by shaking flask experiments in an incubation shaker (Infors Ecotron, Infors, Switzerland) with a batch size of 75 g, a total solids loading of 12% (w/w), and a temperature of 50 °C. The pH of the samples was adjusted with KOH to 5.2, after which 58 mM citric acid/citrate buffer and enzyme were added. The enzyme used was Cellic CTec3 HS (Novozymes, Bagsværd, Denmark), and the dosage of enzyme was 4% (w/w) on substrate total solids. Shaking was set at 250 rpm for the first four h and then at 150 rpm for the following 72 h.

The batch was sampled at 0, 4, 24, and 48 h, after which the samples were diluted ~ 10 times, and the solid phase was separated through centrifugation. The monosaccharide concentration in the supernatant, filtered through a 0.2-µm nylon membrane (Millipore), was then determined through high-performance anion-exchange chromatography (HPAEC). A Dionex ICS-6000 system (Sunnyvale, CA, USA) equipped with a 4 × 250 mm separation column and a 4 × 50 mm guard column (both CarboPac PA1, Dionex) and pulsed amperometric detection were used for this analysis.

The final sample from the hydrolysis (72 h) was centrifuged without prior dilution, and the hydrolysis liquid was stored frozen for further fermentation testing.

### Fermentation

To assess the inhibitory effect of pretreatment liquids on yeast, fermentation tests were performed with the industrial *Saccharomyces cerevisiae* strain Ethanol Red (Fermentis, Marcq en Baroeul, France) using fermentation of a synthetic glucose solution as a reference. All hydrolysis liquids were supplemented with 0.5 mL of a nutrient solution containing 150 g/L yeast extract, 75 g/L (NH_4_)_2_HPO_4_, 3.75 g/L MgSO_4_⋅7 H_2_O, and 238.2 g/L NaH_2_PO_4_⋅H_2_O, and the initial pH was adjusted to 5.5 before inoculation. Freeze-dried Ethanol Red was rehydrated by suspending it in sterile water five times its weight for 30 min at 35 °C, and 1 mL of the suspension was then added to the medium to an initial cell concentration of 2 g/L. The fermentations were run in 30-mL glass flasks containing 25 mL of yeast culture and were agitated with a magnetic stirrer. The flasks were sealed with rubber plugs pierced with cannulas to remove carbon dioxide and were incubated for 48 h in a heating chamber at 180 rpm and 30 °C.

Sampling was conducted after 0, 4, 24, and 48 h, and the ethanol concentration was measured with an Agilent 1260 Infinity high-performance liquid chromatography (HPLC) system (Santa Clara, CA, USA) equipped with a refractive index detector, an autoinjector, and a column oven. An Aminex HPX-87H column and a 125-0131 Standard Cartridge Holder guard column, supplied by Bio-Rad Laboratories AB (Solna, Sweden), were used for separation at 55 °C. The temperature of the detector was set to 55 °C. The eluent was 0.005 M sulfuric acid, supplied at a 0.6 mL/min flow rate.

Initial and residual sugar concentrations were determined with the HPAEC system, Dionex ICS-6000, with CarboPac SA-10 guard and analytical columns.

## Results and discussion

### Extraction with water

The steam-exploded materials were extracted with water to investigate the effects of the addition of 2-naphthol. The phenolic content in water extracts is mainly derived from lignin, whereas carbohydrate degradation gives rise to substances such as furans (including furfural and 5-HMF) and aliphatic carboxylic acids, which, in addition to lignin, affect the aromatic content. The extract potentially contains inhibitory compounds that are detrimental to the growth of microorganisms and to the efficiency of enzymes and consequently diminish ethanol yields [15].

**Fig. 2:**
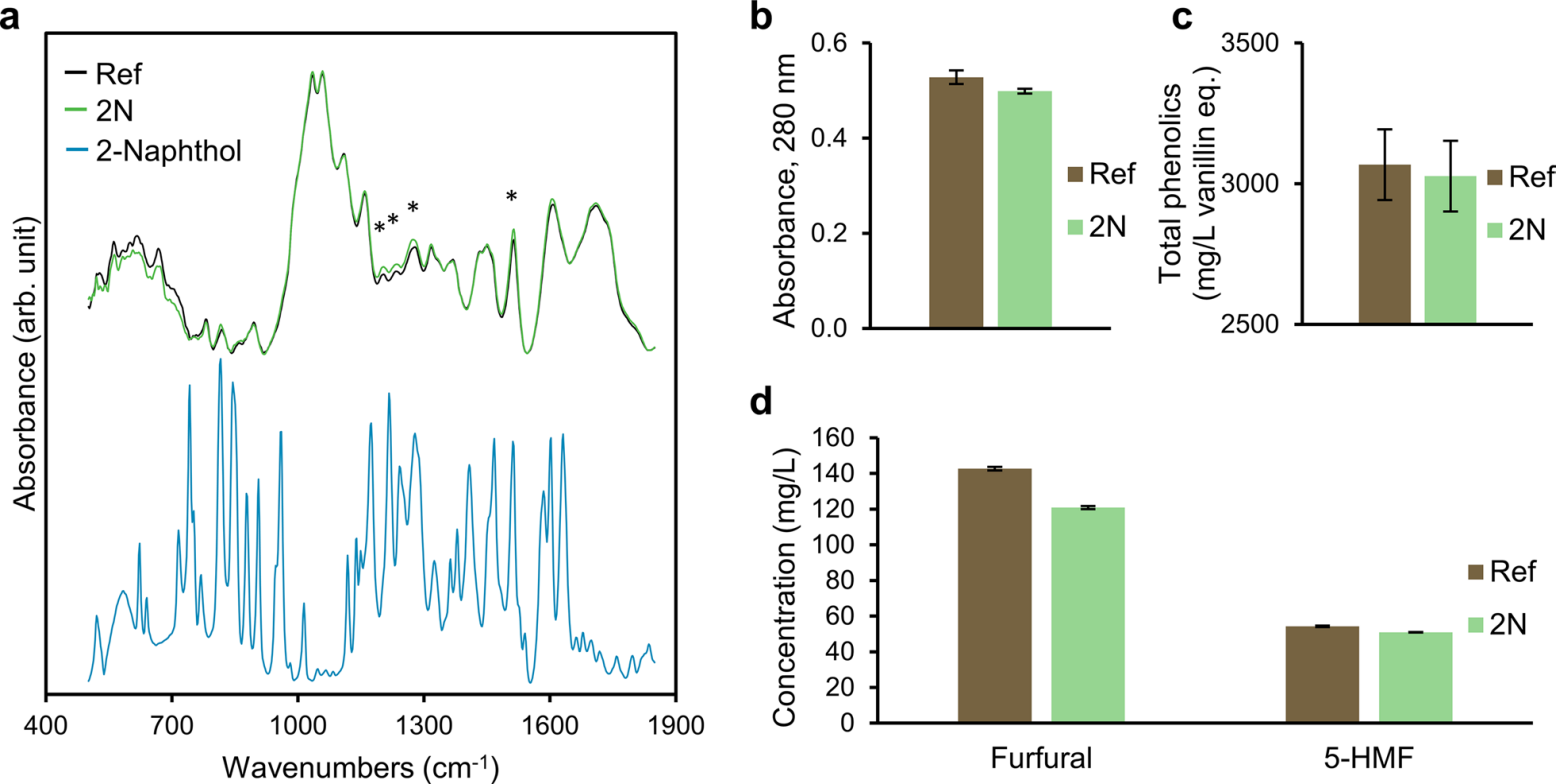
Cut and area-normalized FTIR spectra of steam-exploded biomass samples washed with water (**a**), as well as total aromatics indicated by UV‒Vis absorbance at 280 nm (**b**), and total phenolic (**c**), furfural and 5-HMF (**d**) concentrations in the water extracts. The contribution of the furans to the total aromatic content was subtracted from the UV‒Vis absorbance at 280 nm

The total phenolic content in biomass samples pretreated with or without 2-naphthol was similar, indicating that scavenger addition did not affect lignin (Fig. 2). However, the total aromatic content, determined from the UV‒Vis absorption at 280 nm, revealed a small but significant difference between the two samples. The furan content was analyzed by reversed-phase HPLC to exclude furans derived from the hemicelluloses from the aromatic content. Furans are often found in high concentrations as sugar degradation products after hydrothermal pretreatment [11]. The 5-HMF and furfural concentrations were similar but significantly (p < 0.05) different (Fig. 2). Sample 2N had a slightly lower concentration of furans, which could indicate a slower dissolution of hemicellulose when 2-naphthol was used in pretreatment. Possibly, this is explained by slight mass and heat transfer issues when the scavenger was provided as a milled powder to the process. However, this was not supported by analyses of pH and carbohydrate content. Treatment at a range of severity conditions would be required to be able to draw further conclusions regarding furan formation related to the presence of 2-naphthol.

Although the furan concentration could partially explain the difference in total aromatic content, it was still significantly (p < 0.05) different even after the absorbance stemming from 5-HMF and furfural was deducted from the total absorbance at 280 nm (Fig. 2), which shows that more aromatics derived from sources other than carbohydrates were extractable with water when the treatment was performed without the carbocation scavenger. If the scavenger had reacted as expected, an increase in water-extractable aromatics would be expected. However, a third source of aromatics, tannins, is commonly present in softwood bark and may play a part in the aromatic content. Tannins may also participate in reactions with 2-naphthol, increasing the complexity of the system. Therefore, in this case, the slight difference in aromatic content cannot be used to draw further conclusions regarding 2-naphthol reactions.

The FTIR spectra (Fig. 2) reveal an intensification of the peaks at approximately 1514, 1273, 1232, and 1203 cm^−1^ for the scavenger-pretreated material, which may indicate more G (guaiacyl) lignin units [27–30]. More G-lignin units could imply that the carbocation scavenger retained the lignin structure to a greater degree. However, no indication of 1,2-disubstituted naphthalenes, indicated by strengthened IR signals at 750 and 815 cm^−1^ [1, 30], could be detected, which contradicts earlier findings on pretreated spruce wood [1]. Thus, drawing conclusions regarding 2-naphthol reactions based on FTIR data is impossible in this case. On the other hand, no distinctive features of pristine 2-naphthol were found in the steam-exploded material after washing with water, implying that its concentration, if present, was below the detection limit.

### Extraction with cyclohexane/acetone and ethanol

**Fig. 3:**
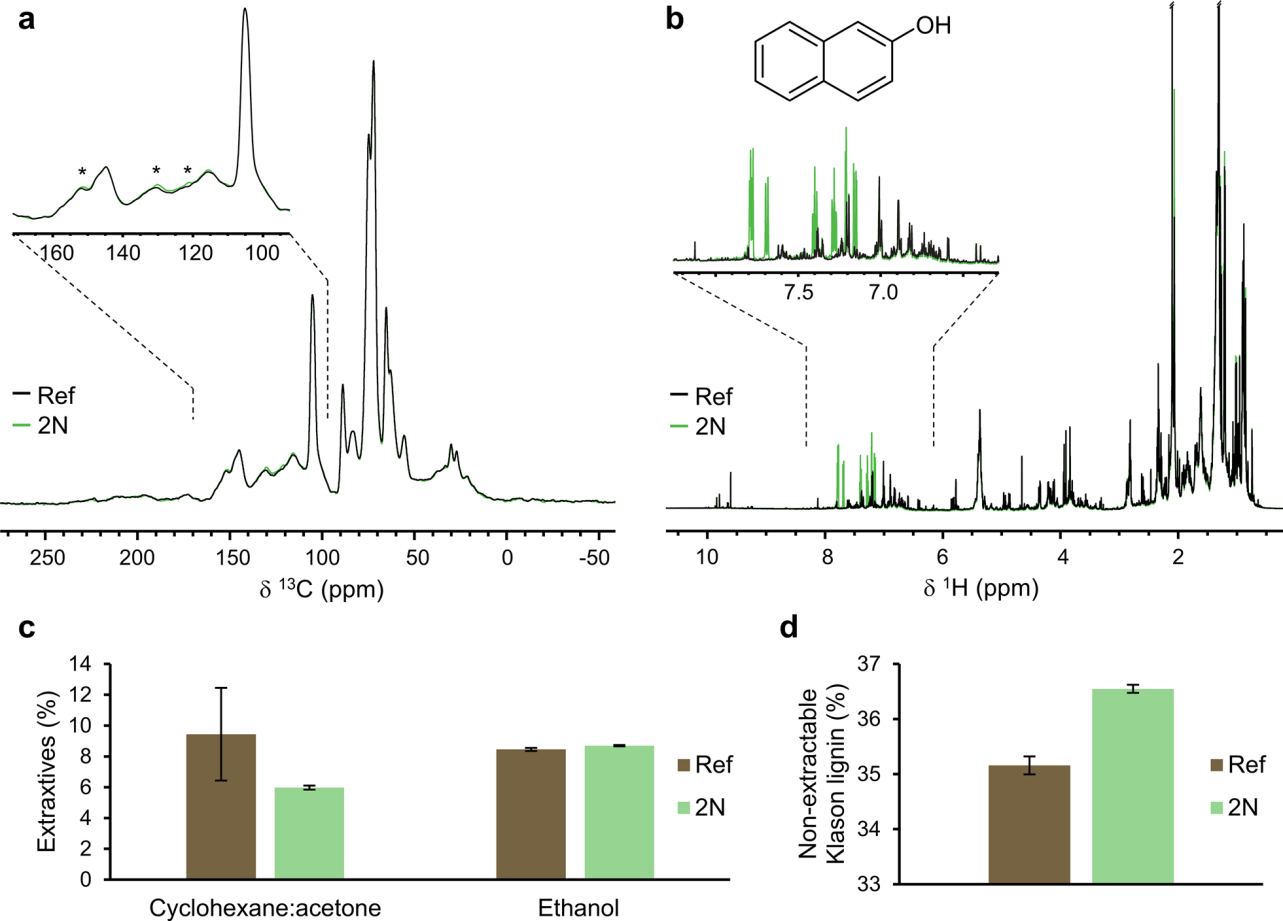
^13^C CP-MAS NMR spectra of steam-exploded biomass, nonextractable with cyclohexane/acetone (**a**) and ^1^H NMR of cyclohexane/acetone extracts (**b**), as well as the extracted mass fractions (**c**) and nonextractable Klason lignin after 2-stage extraction (**d**)

Ground and sieved bark samples Ref and 2N were sequentially extracted with a cyclohexane/acetone mixture followed by ethanol as a polar solvent. The extraction of the reference sample with cyclohexane showed high variability, with extraction yields ranging from 5.8 to 13.0% (Fig. 3). Industrial bark is a heterogeneous material, but as the bark was milled and mixed well before the experiments, the variation is surprising. Approximately 6.0% of the extractives were removed from sample 2N with the nonpolar solvent mixture. The extractions with ethanol did not show the same variability for the two samples. Ethanol removed 8.5–8.7% of the polar compounds from the bark samples, with a slightly greater fraction extracted from the reference sample. Overall, this study did not reveal a higher extraction yield for the material pretreated with 2-naphthol, as previously shown for autocatalyzed spruce [1]. This may be explained by differences in solvent choices and extraction procedures.

The fraction of Klason lignin in the nonextractable residue is displayed in Fig. 3. The sample treated with 2-naphthol had a higher content of acid-insoluble lignin, which could be due to the inclusion of 2-naphthol in the lignin structure by integration of naphthalene rings, as previously shown in the literature [1, 7].

Figure 3 also shows ^13^C CP-MAS and ^1^H NMR spectra of the nonextractable residue and dissolved extracts from cyclohexane/acetone extraction. The ^13^C spectra revealed a slight increase in the intensity of the peak in the aromatic region at 115–156 ppm [32] for sample 2N. Whether this is caused by pristine 2-naphthol remaining in the solid structure or lignin substituted with 2-naphthol cannot be concluded with the current resolution. In contrast, the ^1^H spectra of the extracts revealed unsubstituted 2-naphthol (7.15–7.8 ppm) in the 2N sample (green in Fig. 3b), indicating that some of the added scavenger remained unreacted in the steam-exploded materials.

### Carbohydrate composition

The carbohydrate composition of the pretreated materials, Table 1, revealed a higher glucan content and lower content of the hemisaccharides (mannan and galactan) in the reference sample compared to the sample treated with 2-naphthol. The content of monosaccharides, however, was identical in the two samples. The slightly higher amount of hemisaccharides in the sample pretreated with 2-naphthol coincides with the lower concentration of degradation products (furfural and 5-HMF).

**Table 1:** Carbohydrate composition of the pretreated materials. The polymeric and oligomeric contents (glucan, mannan, and galactan) are reported based on anhydro monomeric mass, while the monomeric contents (glucose, mannose, and galactose) are reported based on the actual monomeric mass

	Ref	2N
Glucan (%)	31.4	28.8
Glucose (%)	0.5	0.5
Mannan (%)	4.2	4.5
Mannose (%)	0.2	0.2
Galactan (%)	1.6	1.8
Galactose (%)	0.6	0.6

### Enzymatic saccharification

**Fig. 4:**
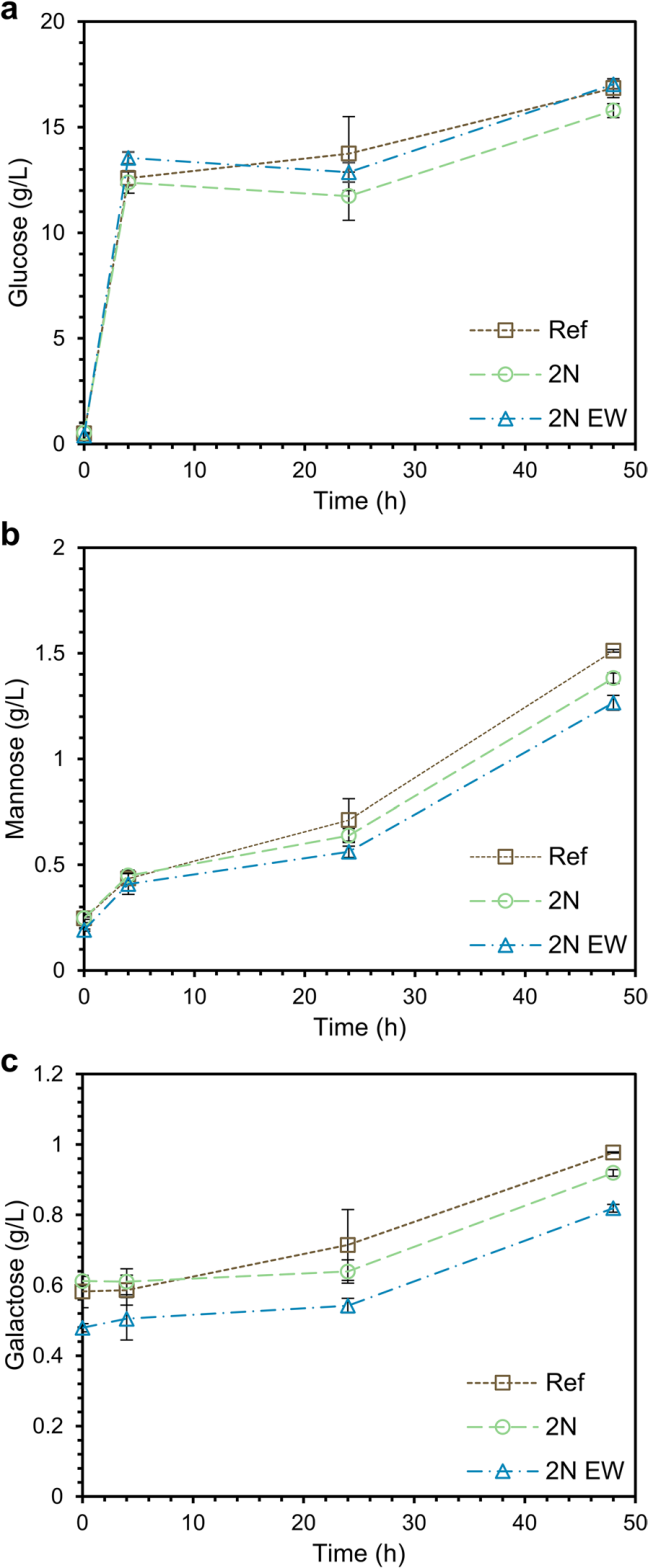
Concentrations of the fermentable sugars glucose (**a**), mannose (**b**), and galactose (**c**) as a function of residence time during enzymatic hydrolysis

The conversion of polysaccharides from bark solids obtained after pretreatment with or without 2-naphthol addition was examined by enzymatic saccharification in a whole-slurry treatment at 12% solids loading. Glucose was the predominant monosaccharide in the hydrolysates, and the concentration was between 15.7 and 17 g/l (Fig. 4a), corresponding to a glucose conversion of 0.39 and 0.40, respectively. The recalcitrance of softwood bark is known from the literature, and its glucose recovery is comparable to other findings [33]. The concentrations of other monosaccharides were much lower than those of glucose and decreased in the following order: xylose, arabinose, mannose, galactose, fructose, and rhamnose. Part of the saccharide content, especially arabinose, stems from pectin fractions typically found in softwood bark [34].

The general trend for the summarized hexose concentrations in the hydrolysates (Fig. 4) was that the reference (Ref) contained the highest concentrations, whereas the hydrolysate from the scavenger-pretreated bark (2N), had a 6.5% lower concentration of hexoses compared to Ref. The total hexose concentrations in the hydrolysate of the 2N EW sample (2N washed with ethanol) were similar to those of the Ref sample. The significantly lower concentrations in the hydrolysate of sample 2N compared to sample Ref is most likely caused by the lower initial glucan concentration in the pretreated substrate (Table 1). In contrast, more glucose was released from glucan in sample N2 EW, which indicates the removal of possibly inhibitory reaction products. Ethanol can remove polar compounds and some nonpolar compounds that may have been attached to the surface of the biomass. The removal of such compounds may, in turn, lead to better accessibility of attachment sites for cellulose-degrading enzymes and possibly less deactivation. In addition, ethanol treatment may have removed water-soluble substances derived as byproducts of the pretreatment process and improved the efficiency of carbohydrate-degrading enzymes [15, 35]. On the other hand, some of the hemicellulose was depolymerized and dissolved during the steam explosion and was inevitably removed during ethanol washing, which explains the lower concentrations of pectic and hemicellulosic saccharides after enzymatic hydrolysis for the detoxified sample (2N EW).

It is evident that 2-naphthol, as a carbocation scavenger, could not be efficiently used for softwood bark in a steam explosion process with the tested setup to improve the enzymatic conversion of resulting substrates at these relatively low severities (severity factor 3.94). This study, together with a preceding study investigating a range of autohydrolysis conditions [18], trials with sulfuric acid as a pretreatment catalyst (data not shown) and enzymatic saccharification conducted on washed substrates (data not shown) unanimously revealed that enzymatic saccharification yields and recoveries did not improve from the addition of a scavenger. However, autocatalytic conditions at higher severities remain to be investigated. Recent research on improved 2-naphthol addition could also unlock yet undiscovered potential in terms of scavenger efficiency for softwood bark treatments [36].

As indicated by the analysis of extracts and nonextractable residues, unreacted 2-naphthol was left to some degree in the steam-exploded material. However, no desired reaction between lignin and 2-naphthol was observed. Although generally considered a useful additive for softwood pretreatment [1, 5, 7, 8], 2-naphthol efficiency could not be proven at severity 3.94 for the treatment of softwood bark. The bark composition, which includes, for example, tannins and pectin [34] at a much higher fraction than in stemwood may have contributed to the challenges of pretreating this material.

### Fermentation

Cultivations of ethanol red yeast using hydrolysates from bark pretreated with or without the addition of 2-naphthol were undertaken to investigate the possible inhibitory effects. Table 2 shows the change in yeast cell mass indicated by the optical density of the fermented broths measured at 600 nm (OD_600_). All the Ref, 2N, and 2N EW cultures remained in the lag phase during the first 6 h of cultivation. The growth phase occurred between the 6 and 24 h samples. Therefore, the exact growth rate in the exponential growth phase could not be calculated due to a lack of observations between 6 and 24 h. However, it appears that cultures from the Ref hydrolysate grew faster than cultures grown on hydrolysate from 2-naphthol-pretreated biomass, judging from the accumulated growth at 24 h. Hampered growth indicates an inhibitory effect that may be attributed to the addition of 2-naphthol, especially due to the presence of pristine 2-naphthol in the fermentation broths. 2-Naphthol is structurally similar to well-known inhibitors, such as phenols, 5-HMF, and furfural [15], and potentially possesses related inhibitory effects.

**Table 2:** Optical density measured at 600 nm (OD_600_) for the fermentation broths and yield of ethanol from the fermentable sugars (glucose, mannose, and galactose) at different fermentation times

	OD_600_ (-)			Yield (g/g)	
	6 h	24 h	48 h	24 h	48 h
Ref	0.062	0.963	1.160	0.45	0.45
2N	0.072	0.796	1.011	0.45	0.46
2N EW	0.056	0.829	1.230	0.49	0.49

**Fig. 5:**
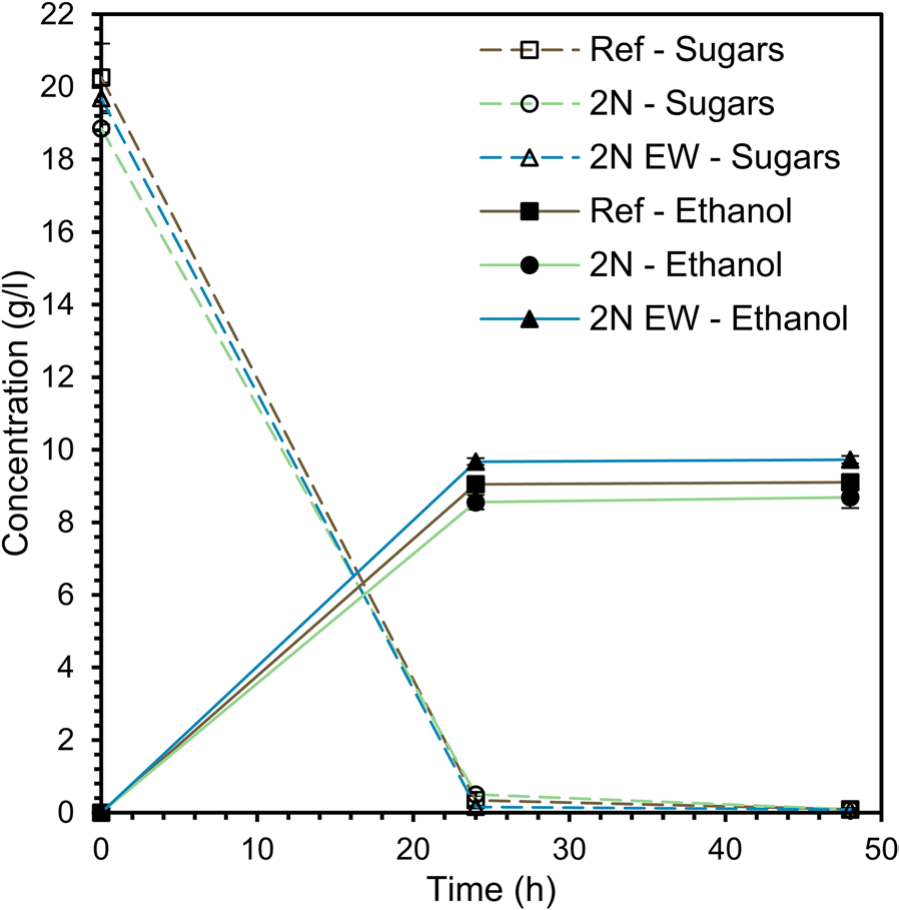
Concentrations of ethanol and fermentable sugars (glucose, mannose, and galactose) as a function of fermentation time

Ethanol release from glucose conversion by the yeast cells was first observed after 24 h (Fig. 5). At the same time, glucose was not detectable in the culture broth. Further yeast growth was based on the consumption of other monosaccharides, such as mannose and galactose, that were present in the enzymatic hydrolysate. Analysis of the culture broth by HPAEC revealed that the yeast consumed mannose, galactose, and glucose. The galactose concentration also decreased from approximately 0.75–0.9 g/L to zero after 48 h and was almost completely consumed after 24 h. Ethanol red yeast has been shown to consume galactose in nutrient-rich media, especially those containing nitrogen [37].

The yield of fermentable sugars (glucose, mannose, and galactose) to ethanol (Table 2) revealed that the fermentation yield was unaffected by the 2-naphthol dosage, although the yeast cell growth was slightly lower. On the other hand, the detoxified sample, 2N EW, performed somewhat but significantly better than the other samples in terms of ethanol yield, reaching close to the theoretical limit of 0.51 g/g. The improved performance confirmed that the ethanol washing procedure could also remove other inhibitors. Previous studies on softwood [6] have indicated a strong cross-inhibitory effect between 2-naphthol and furans and possibly other compounds such as organic acids. This study showed that inhibition can be overcome by selecting pretreatment conditions, such as mild pretreatment conditions and a continuous process where furfural is continuously stripped from the substrate. However, under mild pretreatment conditions, the potentially positive effect of scavenger addition may not be adequate. Considering the growing number of published works utilizing 2-naphthol pretreatments for the enzymatic conversion of biomass [1–10, 38], surprisingly few studies have investigated fermentation to target an actual market product. This study highlights the necessity for jointly optimizing all the main process steps since 2-naphthol may have a beneficial effect on hydrolysis yields depending on the raw material, pretreatment severity, and dosage, which in turn, due to cross-inhibition, has a contradictory impact on fermentation recovery if bioethanol is the intended product.

## Conclusions

This study showed that the carbocation scavenger 2-naphthol is not a useful pretreatment additive for the production of sugars or bioethanol from softwood bark via a continuous and industrially scalable steam explosion process at severity factor 3.94. A lower initial growth rate indicated the yeast growth was slightly inhibited upon scavenger addition. Moreover, no beneficial effects on sugar recovery from enzymatic hydrolysis were detected. However, ethanol yields after 24 h were not negatively affected by scavenger addition, showing that cross-inhibitory effects can be overcome if the correct parameters for the pretreatment process are selected. Furthermore, a simple detoxification procedure was demonstrated, showing that ethanol yields can be improved further by washing the substrates with ethanol before enzymatic hydrolysis and fermentation, which likely removes 2-naphthol together with other inhibitors. Overall, this information may prove helpful when investigating carbocation scavenger treatments for different raw materials and highlights the importance of jointly investigating and optimizing all the main process steps.

## Data Availability

The datasets used and/or analyzed during the current study are available from the corresponding author upon reasonable request.
